# A Case of Herpes Zoster

**Published:** 1869-03

**Authors:** F. B. Greenough

**Affiliations:** Boston


### A CASE OF HERPES ZOSTER.

Read before the Boston Society for Medical Observation, by F. B. GREENOUGH, Boston.

The following case of herpes zoster nuchæ, vel collaris, accompanied by, or coinciding with, a facial paralysis on the same side, occurred in the practice of Dr. John Homans, and it is through his kindness that I have been enabled to see and report it. It has been pretty universally conceded that herpes zoster is often, in some way, connected with a lesion of the cutaneous nerves of that portion of the integument over which it is distributed. The reasons for assuming this may be briefly stated as follows: The eruption always seems to follow the course of a nerve; this is constant, and, although there may be more or less irregularity in its distribution, on the whole, the course of the eruption coincides pretty nearly with that of the nerve. Moreover, the eruption is very often preceded, accompanied and sometimes followed by, neuralgic pains in the nerves over which it appears. That these pains are really of a neuralgic character, and not merely due to the soreness of the eruption itself, is proven, first, by the character of the pain; and second, by the fact that the pain not only often exists before the eruption appears, and continues after it has disappeared, but that it is even worse sometimes at that time, than while the eruption is present. As has been said, then, it is admitted that herpes zoster is in some way connected with an affection of the nerves, as is shown by the coëxistence of neuralgic pain; that is to say, by an affection of the sensitive fibres of the nerves. *A priori*, we should suppose that any influence or cause that was able to produce an abnormal condition of the sensitive fibres of a mixed nerve, would also affect the motor fibres. Thus, we know that in a bad case of sciatica, the leg is for the time being more or less paralyzed. I have not, however, been able to find any mention of a motor paralysis in connection with herpes zoster, except in Hebra.* He states:—"It sometimes happens that the persistence and intensity of this symptom" (*i.e.*, neuralgia) "render the disease a very painful one; and in some cases the functions of the motor nerves also are interfered with."

That Hebra makes such a statement as this, is sure proof that he not only has seen cases where a paralysis of motion coëxisted with zoster, but that he also convinced himself that there was some connection between the two, as he is not apt to take anything on hearsay, nor to believe what he has not seen. There are reasons why in many cases of zoster, even if a lesion of the motor fibres did exist, it should have escaped notice. The most common situation of zoster is over one or more of the intercostal nerves, whence its synonym, zona. If we reflect on the nature and use of the intercostal muscles, whose motive power depends on these nerves, we shall see that one or two of them might be paralyzed without causing noticeable inconvenience. For we cannot voluntarily use them separately; and even if one or two did not contract, the others would be sufficient to carry on the respiration. When zoster is situated on the legs or arms, it is distributed over the course of the larger cutaneous nerves, whose motor fibres also might be paralyzed without causing any symptom. In zoster cervicalis, however,

* Hebra on Diseases of the Skin. Translated by Fagge. New Sydenham Society. London. 1866.

the case is different, as will be shown later. It is on account of a symptom which theoretically seems naturally to belong to a case of zoster, having escaped the observation of all writers on the subject except Hebra, that the following case has been thought worth reporting:—

W. K., æt. 33; married; mechanic. Patient has always enjoyed fair health up to about a year ago, although he has never been very robust. Some time in December, 1867, he had an acute affection of the lungs, for which he came under the care of Dr. John Homans. The diagnonis was pneumonia. He was ill about eight weeks, and made a slow recovery. Ever since that time he has been troubled with a cough, some pain in his chest, muco-purulent expectoration, loss of flesh, night-sweats, etc. In short, he has had the rational signs of tubercular trouble. During the last part of October, 1868, he had considerable pain, of a neuralgic character, about the left side of the face and neck. No history of exposure to cold could be obtained.

On the 1st of November, an eruption appeared on the left side of his neck. From his description, the eruption appears to have been vesicular, as he says it was covered with "blisters," some of which he pricked, and a clear, transparent fluid oozed out. There was no itching, but much smarting and burning. The neuralgic pain continued, but was somewhat less than before the eruption made its appearance. On November 11th, he first noticed what he describes as a numbness of the left side of his face. He could not close his left eye, his mouth was drawn to the right side, and he was much troubled by his food getting between his gums and his left cheek, from whence he had to continually remove it with his finger. There was no loss of motor power of the extremities, nor, as far as could be learned, was there any abnormal condition of sensation of any part of the body. After a day or two, the left eye became injected and painful.

I first saw the patient on November 19th; that is to say, on the nineteenth day of the eruption, and the eighth of the facial paralysis. He was a man of medium size, thin and pale, with an occasional hacking cough. No examination of the chest was made, but I have been informed by Dr. Homans that there is undoubtedly some solidification of the lower part of the right lung. The eruption on the left side of neck consisted of several patches about the level of the thyroid cartilage, and extended backwards and upwards. The patches were more or less oval, varied in size from one-fourth to one inch in diameter.

They were separated from each other by intervals of healthy integument. They consisted of red, inflamed bases, being covered partially by dirty yellowish crusts; and, where these were wanting, showing small, superficial, but angry-looking ulcerations. Each patch was surrounded by a well-marked red areola. One of the patches extended to the median line, in front, at level of thyroid cartilage; another reached almost to the median line behind, a little below the occipital protuberance; and there were some behind the ear. No vesicles or pustules could be seen; but it seemed probable, from the appearance of the crusts and ulcerations, that the former had been produced by the drying up of the vesicles and pustules, and the latter by their destruction. The left eye could not be closed, the left nostril was flat, and the mouth was drawn to the right side. The tongue was protruded straight, the uvula hung perpendicularly in the median line, and on saying ah! no one-sided action of the muscles of the soft palate could be detected. The conjunctiva of left eye was normal, but, by patient's account, it had been quite inflamed up to two days previous. Both pupils reacted normally to light, and were of equal size. Sensation of left side of face was normal, and on a careful examination, no evidence of any impairment of motion or sensation of any other part of body could be found. There was no discharge from the left ear. Patient stated that he could not hear as well with his left as with his right ear, and on testing with watch the power of perception of sound, did seem less on that side. The test, however, was not very satisfactory, nor could a definite answer be got from the patient as to the existence or not of this deafness previous to present illness. There was still some neuralgic pain about left side of face and neck, but much less severe than at first. There was not, and never had been, any twitching of left side of face.

After this, the course of the disease was steadily towards cure. The ulcerations healed, the crusts fell off, the pain diminished, and the power of motion slowly returned to the facial muscles. The improvement, in this latter respect, was so gradual that it was not noticed by either the patient or his wife, from day to day. At present, his face is perfectly natural. The eruption lasted about four weeks, and left scars behind it. The paralysis lasted a little longer.

Such, very briefly stated, is the case which I have thought worth reporting. For a consideration of it, three series of investigations suggest themselves:—

1st. As to the nature of the eruption.

2d. As to the nature of the paralysis.

3d. As to the nature of the connection between the two.

With regard to the nature of the eruption. Hebra's* definition of herpes is as follows:—"It is benign, runs an acute course, and is attended with the formation of miliary papules, which are arranged in groups, and generally undergo development into vesicles and pustules as large as lentils, or even still larger. It is never distributed over large tracts of the cutaneous surface, being always confined to certain definite regions. After remaining a few days, or as long as four weeks, it dries up into flat crusts, which often leave scars when they fall off." This definition applies to the whole genus herpes, one of the species of which is herpes zoster, which he describes as follows:—"I include under this name all those skin affections which present the characters of herpes, and in which the part of the surface occupied by the groups of vesicles corresponds to the distribution of certain cutaneous nerves, and which last (whether occurring on the head, trunk, or limbs) are confined to one-half of the body."† To the species zoster he gives different names, according to the part of the common integument on which the eruption is developed. One of the local varieties is thus described, under the name herpes zoster nuchæ, vel collaris:—"In this form of shingles the eruption first makes its appearance on the side of the neck, over the second and third cervical vertebræ, and extends thence upwards towards the lower jaw and face, forwards towards the larynx, and lastly downwards; a few clusters reaching even as far as the second rib."‡

It must be seen how perfectly these definitions apply to the case under consideration. It is true that, when seen by me, no vesicles were present, but the appearance of the crusts and ulcerations, on the nineteenth day of the eruption, was such as to convince any one that vesicles and pustules had existed, without falling back on the history of the case, as given by the patient himself. The situation of the eruption was over the second and third cervical nerves and their branches; two patches or groups of crusts were over the course of the superficialis colli; others were over the auricularis magnus and occipitalis minor, and others still over the occipitalis major. The course of the disease tallies exactly with Hebra's definition: the efflorescence was unilateral, coming up sharp to the median line in front, and nearly so behind; and, moreover, the characteristic neuralgic pain of herpes zoster was present. If this positive

* *Op cit.*, page 368. † *Op cit.*, page 372. ‡ *Op cit.*, page 376.

evidence is not enough, we have the negative evidence of exclusion. The only other cutaneous diseases, except herpes, to which an eruption having this location and appearance could belong, are eczema, sycosis, and some of the syphilodermata.

That it was not an eczema, is shown by the absence of itching, by the presence of ulcerations, by the large size of the vesicles, as described by the patient and proven by the size of the crusts, and, lastly, by the fact of a scar being left behind after the eruption had healed.

To exclude sycosis, we have the existence of patches beyond the region of the beard, as behind the ear, the sharply-defined character of the patches, with healthy skin between, the absence of any evidence of inflammation of the hair-follicles, and, lastly, the spontaneous cure in four weeks.

A syphilitic eruption with crusts and ulcerations would have had thicker crusts and deeper ulcerations, and other evidences of the presence of the syphilitic virus in the system would probably have been found.

Assuming, then, that the eruption was herpes zoster, let us glance at the paralysis, and see if any conclusion with regard to its nature can be arrived at.

From the muscles that were paralyzed, *viz.*, the left orbicularis palpebrarum, the levators of the upper lip and nostril, the left side of the orbicularis oris, and the left buccinator, the nerve affected must have been the left facial or seventh cranial nerve.

Facial paralysis is one of the most common lesions of motor function observed. Like other nervous lesions, it is generally divided by writers into two classes, the organic and functional; the former consisting of those cases in which some organic disease of the nerve or nervous centres exists, and the latter of those in which all we can get evidence of is an abnormal state of functional activity, without appreciable cause.

This classification is not a very satisfactory one, as when we call a paralysis functional, we simply state in other words that we know nothing about it. There is some reason in speaking of organic and functional diseases of the heart and stomach, for instance, as we see the functions of these organs often interfered with, while the organs themselves are perfectly healthy; as, for example, palpitation from excitement, or arrest of digestion from anger or fear. In such cases, we know that there is no disease of the organs, and fall back on the nervous system as being the cause of the trouble. But in case of derangement of the nerves themselves, we have nothing to fall back upon, and we must consider that perfect integrity of nerve tissue must be accompanied with normal functional activity. There certainly are cases where we do not, and probably never shall, know what the lesion is, but that it exists must be admitted. Undoubtedly, half a century ago certain nervous affections would have been classed as functional, which now are shown by the microscope to be due to a hypertrophy of the connective tissue of the neurilemma, and a consequent pressure on the nerve fibres. The term, however, is a convenient one, if it is only used understandingly.

An organic paralysis of the facial nerve might be due to many causes, such as any disease of the encephalon or nerve itself, or pressure, as by blood, serum, or pus, or by a tumor of the brain itself, or of the meninges, by an exostosis, or by a depressed fragment of a fractured skull; or, in short, by any cause which should produce pressure on the brain or nerve.

This pressure might be situated either on that part of the nerve which lies within the cranium, or within the aqueductus Fallopii, or after it has emerged from the styloid foramen. Any destruction of continuity of the nerve would also cause paralysis. In the case under consideration, the whole series of intracranial lesions is excluded, by the fact that no other cranial nerves were affected, nor was there any paralysis of the spinal nerves distributed to the extremities. Had the trouble been in the Fallopian canal, it probably would have been due to caries of the bone, in which case we should have some symptoms, as pain, otorrhœa, etc. It has been noticed in many cases of hemiplegia, that one side of the tongue and half the uvula and soft palate are paralyzed. This is due to the fact that the facial nerve is connected with the gustatory nerve, by means of the chorda tympani, and also furnishes motor fibres to the azygos uvulæ and muscles of the soft palate through the large petrosal nerve, which goes to the spheno-palatine or Meckel's ganglion, from which the motor fibres descend to the above-mentioned muscles. Both these branches are given off from the facial within the aqueduct of Fallopius; and consequently, had there been any pressure or disease there, the tongue and uvula would have been paralyzed on that side. That there was no tumor pressing on the nerve after its exit from the styloid foramen, was evident through palpation and inspection. Moreover, the course of the disease was not that of an organic lesion. It came on suddenly, it is true, by the patient's account; but it only lasted about five weeks, and disappeared entirely. It may have come on so slowly that the patient may not have noticed it until it was complete. This seems more than probable, when we remember that that was the way in which it disappeared.

Having, then, excluded organic lesions, in the common acceptation of the term, let us see by what other causes facial paralysis may be produced. Most writers on the subject consider that this affection is often due to the effect of cold or damp air, especially as a draught. Niemeyer, Grisolle, Brown-Séquard, and Flint, who certainly stand at the head of the ranks of medical writers in their respective countries, all agree in ascribing to this cause the great majority of cases of uncomplicated, functional, facial paralysis. One thing should be kept in mind, and that is, that of all the causative influences to which disease is ascribed, that of cold is one of the most unsatisfactory. It is an influence which, living as we do, can almost always be found, as few persons can look back for a few days without recollecting some occasion on which they were exposed to a draught of wind, or had gone from a high temperature into a lower one.

It is not meant to deny the influence of cold in sometimes causing disease; but every physician knows how often the formula "taken a little cold" is used to represent some entirely unknown pathological condition. Besides, in this case, strange to say, the patient could not refer to any particular exposure. Let us look farther, then, and see if some more definite diagnosis cannot be arrived at.

There is a class of functional or essential paralyses, about which much has been written. I refer to the so-called reflex paralysis; that is to say, to those cases in which paralysis follows peripherical irritation. Brown-Séquard, in his "Lectures on the Diagnosis and Treatment of Functional Nervous Affections,"* places at the head of the list of the causes of functional affections of the nerves," an irritation (by worms, by teething, or a decayed tooth, by a cold, by a burn), a wound, an inflammation, a neuralgia, etc., of centripetal fibres."

The fullest and most perfect account of these cases is that given by Dr. Graves, in a course of lectures given at the Meath Hospital at Dublin. I cannot do better than to quote an abstract of the lectures referring to this subject, as given by Dr. Graves in a later publication.† He says: "In the lectures to which I have already referred, I showed that this mode of accounting for all forms of paralysis, by referring them to original disease of the nervous centres, was in many cases incorrect, and proved, I think to the satisfaction of the class, and those who read the lectures, that a most important and influen-

* Page 15. † System of Clinical Medicine. Dublin. 1843. P. 396.

tial cause of paralysis had been hitherto nearly overlooked; a cause which, commencing its operation on the extremities, and not on the centres of the nervous system, might, by a reflex action, produce very remarkable effects on distant parts. I brought forward on that occasion many arguments, facts, and cases, to prove the possibility of such an occurrence, to show that it frequently happens that impressions made on the extremities of nerves will generate a morbid action in them; that this morbid action will be conveyed along their branches and trunks, to the spinal cord, or brain; and that, continuing its propagation, it may, by a retrograde course, be carried thence along the nerves to distant organs, and in this way give rise to disease in parts originally intact and healthy. I brought forward several instances to prove that when a certain portion of the extreme branches of the nervous tree has suffered an injury, the lesion is not confined merely to the part injured, but in many cases is propagated back, towards the nervous centres; and that in this way, not only the nervous filaments of the injured part may be affected, but also the main trunk of the nerve and other branches; or that the lesion may reach the brain, or spinal cord, and thus produce still more extensive effects on the system. What I endeavored to impress upon the class at that time was, that pain, numbness, spasms, and loss of power of muscular motion, may be produced by causes acting on the extremities of the nerves; and that such affections, commencing in the extremities of the nerves, may be propagated towards their centres, so as to be finally confounded with diseases originating in the centres themselves."

Many interesting cases are given, all of which go to show that paralysis of motion has been observed following, or coinciding with, the most different species of irritation of the periphery of the nerves. I will only quote one. "A young lady having wounded the inside of her ring-finger with a blunt needle, observed that she had, in consequence of the injury, a considerable degree of numbness, not only of the finger wounded, but also of the little finger next it. Here we find that an impression, made on the nerve of one finger, not only affects that finger, but travels backwards, so as to operate on the branch given by the ulnar nerve to supply that finger, and given off, observe, above the place of the wound, so that the phenomena were identical with those which would arise from an injury inflicted on the branch which would supply both fingers."*

Flint, on the other hand, is inclined to doubt the existence of

* *Op cit.*, p. 397.

reflex paralysis. He says:—"Cases have been reported by Graves, Romberg, Rayer, Brown-Séquard, and others, within late years, of paraplegia, apparently referable to various affections of different organs—*viz.*, the kidneys, bladder, uterus, ovaries, intestines, etc. A causative connection between the paralysis and the diseases seated in these organs, is inferred, from the fact that recovery from the former takes place after the latter are cured. It is supposed that the local diseases induce paralysis, by a morbid influence transmitted through the reflex system of nerves; and hence the term reflex paraplegia has been used to distinguish the affection in these cases. In order to establish a pathological connection between different local diseases and paralysis, it is necessary to show that the former precede the development of the latter, in a proportion of cases too large to be explained by merely accidental coincidence. It is questionable whether facts sufficient to show this have been yet accumulated. And when, on the other hand, it is considered that the various local diseases supposed to be adequate to the causation by a reflex influence, are not accompanied by paralysis in the vast majority of cases, the existence of a causative relation may reasonably be doubted in the cases in which the association exists."*

It must be confessed that there is some force in this argument. Let us see if the anatomical relations of the nerves affected in this case are such as to warrant the diagnosis of reflex paralysis, assuming that such a disease does exist.

The facial nerve derives its roots from the floor of the fourth ventricle, and from the groove between the olivary and restiform bodies. The posterior roots of the cervical nerves which contain the sensitive fibres, of whose lesion we have evidence, arise from the posterior columns of the cord. These points of origin are not so distant but that, could no other more plausible theory be found, we might suppose that the paralysis was due to a morbid influence transmitted along the cervical nerves, and caused by the eruption, to the cord, and thence propagated outward along the facial. Would not, however, the tongue and uvula be paralyzed, if the cause of the paralysis was sent out from the origin of the nerve?

While searching for the possibility of a connection between the roots of these nerves, we must not neglect to see if they are connected together at their periphery; and, in so doing, we find a most intimate connection by means of anastomoses.

These anastomoses, which connect the second and third cer-

* Principles and Practice of Medicine. Philadelphia. 1867. P. 635.

vical with the facial nerve, are three in number. 1st, the superficialis colli joins a descending branch of the facial; 2d, the auricularis magnus joins the facial by quite a large branch; and 3d, the occipitales major and minor inosculate with the posterior auricular. A glance at the case under consideration shows us that these very three branches of the cervical plexus, which have such an intimate connection with the facial nerve, are the ones over which the herpetic eruption was developed. We have, then, here an affection of two different nerves, which are anatomically very intimately connected at their peripherical ends; the lesion of the one being manifested by a paralysis of motion, and this is the only symptom we can expect, as the nerve consists only of motor filaments; that of the other being shown by neuralgic pain, but we cannot say but what the motor fibres also are affected, the distribution of the nerve being such, that if they were, no symptom would be given. Is it not logical to assume that these two affections spring from a common cause? What the cause may be we do not know, but we do know that for some reason, in most cases of zoster, we have a lesion of certain nerves; we know that to be so in our case; but, in addition, we find a lesion also of another nerve, which is intimately connected with the first.

It certainly seems more natural to ascribe these both to a common cause, than to go to reflex paralysis (which, perhaps, does not exist as a disease) for a solution. What could the possible connection between these two coëxisting affections be?

They might be simply the accidental result of coincidence.

They might stand to each other in the relation of cause and effect.

Or they might both be due to a common cause.

The intimate anatomical connection between the two nerves, makes it more than probable that there is something more than mere coincidence. The zoster could not be caused by the paralysis, as it preceded it; the paralysis might have been caused by the zoster, if reflex paralysis is admitted, and if enough has not been said to exclude it. Or, they might both be due to some common cause; as we suppose, some unknown lesion of the nerves themselves.

One other influence at whose door the causation of both lesions might be laid, should be mentioned, namely, cold.

It has already been stated that many cases of facial paralysis are supposed to be due to this agent, and by several writers a prominent position is given to cold in the etiology of herpes zoster.

I can only repeat what has been said, as to the unsatisfactory evidence of cold, as a cause of disease, especially where, as in this case, there is no evidence of any exposure to it.

I have endeavored to show that in this case of zoster, we have evidence of other nervous lesions besides neuralgia, which, if corroborated by other cases, will certainly show that the unaccountable neuralgic pain which so frequently accompanies zoster, is due to some lesion of the nerve itself.

Of course nothing has been proved, and undoubtedly this will pass with many as a case of reflex paralysis. Whether such a disease exists or not, I do not pretend to decide; but I have endeavored to show that this case is probably not an example of it.

Be this as it may, however, it has at least been shown that there probably is some connection between the zoster and facial paralysis in this case; and should other similar cases be noticed and reported, perhaps some light might be thrown on what is at present, as far as pathology and etiology go, a most obscure disease.

If this brief paper should be the means of calling the attention of those of the profession, who have the opportunity of seeing much cutaneous disease, to this point, it will accomplish all that the writer dares to hope for.—*Boston Medical and Surgical Journal.*

